# A Novel *in Vivo* Model for Assessing the Impact of Geophagic Earth on Iron Status

**DOI:** 10.3390/nu8060362

**Published:** 2016-06-13

**Authors:** Gretchen L. Seim, Elad Tako, Cedric Ahn, Raymond P. Glahn, Sera L. Young

**Affiliations:** 1 Division of Nutritional Sciences, Cornell University, Ithaca, NY 14853, USA; gs373@cornell.edu; 2 USDA-ARS, Robert W. Holley Center for Agriculture and Health, Ithaca, NY 14853, USA; et79@cornell.edu (E.T.); Raymond.Glahn@ars.usda.gov (R.P.G.); 3 Department of Food Science, Cornell University, Ithaca, NY 14853, USA; cahn954@gmail.com; 4 Department of Population Medicine, Cornell University, Ithaca, NY 14853, USA; 5 Department of Anthropology, Northwestern University, Evanston, IL 60208, USA

**Keywords:** geophagy, iron absorption, intestine, broiler chicken, anaemia, pica, clay

## Abstract

The causes and consequences of geophagy, the craving and consumption of earth, remain enigmatic, despite its recognition as a behavior with public health implications. Iron deficiency has been proposed as both a cause and consequence of geophagy, but methodological limitations have precluded a decisive investigation into this relationship. Here we present a novel *in vivo* model for assessing the impact of geophagic earth on iron status: *Gallus gallus* (broiler chicken). For four weeks, animals were gavaged daily with varying dosages of geophagic material or pure clay mineral. Differences in haemoglobin (Hb) across treatment groups were assessed weekly and differences in liver ferritin, liver iron, and gene expression of the iron transporters divalent metal transporter 1 (DMT1), duodenal cytochrome B (DcytB) and ferroportin were assessed at the end of the study. Minimal impact on iron status indicators was observed in all non-control groups, suggesting dosing of geophagic materials may need refining in future studies. However, this model shows clear advantages over prior methods used both *in vitro* and in humans, and represents an important step in explaining the public health impact of geophagy on iron status.

## 1. Introduction

Geophagy, the intentional ingestion of earth, is practiced in many cultures on all inhabited continents [1,2,3]. Despite widespread consumption and documented potential for both positive and negative health consequences, the etiology and consequences of geophagy remain unclear [4]. Geophagy has been frequently associated with anemia, especially iron (Fe) deficiency anemia. Anemia remains a significant global health concern. As much as 8.8% of the total disability from all conditions in 2010 was attributed to anemia, the most common etiology being Fe deficiency [5]. Pooled analysis of the relationship between geophagy and anemia revealed that geophagy is significantly associated with lower blood hemoglobin (Hb) levels (weighted mean difference = −0.95 g/dL) and individuals with geophagy were two times more likely to be anemic [6]. Because of this relationship, the majority of explanations about the causes and consequences of geophagy involve Fe [3,7,8,9,10,11,12,13,14]. One explanation holds that geophagy is an adaptive response to Fe deficiency, *i.e.*, it is a means to increase Fe intake [15,16,17]. Another is that geophagy is a non-adaptive response to Fe deficiency; altered brain function has been speculated to cause cravings for earth and other non-food items [18]. The causality has also been posited in the opposite direction: geophagy may cause Fe deficiency by inhibiting its absorption [3,9]. This may occur through dietary Fe being adsorbed by the earth in the alimentary canal or by earth binding to the mucin layer of the intestine, creating a physical barrier to Fe absorption [19,20,21,22].

A number of studies have attempted to elucidate the relationship between Fe status and geophagy through *in vitro* analysis of geophagic substances. Data on total elemental composition (TEC) have been the focus of the majority of the analyses of geophagic earth performed to date [7,15,23,24,25]. Most of these studies have reported high concentrations of Fe in geophagic earth relative to concentrations found in food, leading the authors to conclude that geophagy is a source of Fe. Yet TEC does not account for bioavailability, which is known to be highly variable depending on the chemical form of the Fe and other factors [26]. A smaller number of *in vitro* studies have taken bioaccessiblity (the fraction of a mineral element that is soluble in the gastrointestinal environment and available for absorption [27]) or bioavailability (the proportion of an ingested mineral element that is absorbed and either utilized in a metabolic pathway or sequestered in body stores [28]) into account. Most [20,29,30,31] but not all [32] found little bioaccessible Fe. Three *in vitro* studies to date have also investigated the potential ability of geophagic earth to adsorb Fe from a food source or Fe in solution, thereby making it unavailable for absorption. These studies concluded that some, but not all, geophagic earth samples were able to significantly adsorb bioavailable Fe, *i.e.*, they have the potential to reduce Fe absorption [20,33,34].

Although these *in vitro* studies provide valuable information about the potential of geophagic substances to affect Fe absorption, alone they cannot definitively answer questions about causality. *In vivo* experiments offer a far better approximation of how geophagic earth impacts Fe absorption, in part because they are able to better capture the interaction of geophagic substances with the mucin layer as well as interactions with other ingesta. *In vivo* studies have actually been conducted in humans with Turkish [19,21,35], Texan [36], and South African [22] geophagic earths and found that geophagy does not increase Fe status and may even decrease it. However, these experiments were done more than 30 years ago and have a number of limitations, including outdated methods, very small sample sizes, and inadequate statistical analysis [9]. Furthermore, the mechanisms by which geophagic substances inhibit Fe absorption as well as the magnitude of the effect on Fe status remain unclear.

Given the limited *in vivo* data about the influence of geophagy on Fe absorption, our objectives were two-fold. The first was to develop an *in vivo* model that would permit the rigorous assessment of the capacity of geophagic earth and clay minerals to influence Fe status. We selected *Gallus gallus* (broiler chicken) as a model, which has been demonstrated to be a useful and sensitive method to test Fe bioavailability from a variety of Fe sources [37,38]. Our second objective was to test the effects of geophagic earth and clay minerals on a range of markers of Fe status. This included blood hemoglobin (Hb) concentration, liver ferritin and liver Fe concentrations, all of which decrease in response to dietary Fe deficiency. Additionally we measured duodenal expression of genes related to Fe absorption (divalent metal transporter 1 (DMT1), ferroportin, and duodenal cytochrome B (DcytB)) which have been shown to increase in response to Fe deficiency. All of these Fe status markers have been shown to be appropriately sensitive to iron status in the broiler chicken model [37,38].

## 2. Materials and Methods

### 2.1. Animals and Diets

One hundred and twenty Cornish cross fertile broiler eggs were obtained from a commercial hatchery (Moyer’s chicks, Quakertown, PA, USA). The eggs were incubated in the Cornell University Animal Science poultry farm incubator. After hatching, chicks were housed in a total-confinement building (three chicks per 1 m^2^ metal cage) under controlled temperatures. The chicks were exposed to 16 h of light daily. Chicks were given *ad libitum* access to water throughout the study and *ad libitum* access to the experimental diet (Table 1) during the day; feed was taken away at night and replaced each morning after gavaging. The experimental diet was formulated to be marginally adequate in Fe so that Fe status would be more sensitive to different Fe intakes. All animal protocols were approved by the Cornell University Institutional Animal Care and Use Committee (Ethic approval code: 2007-0129).

The geophagic earth used in this study, which has been previously characterized by Seim *et al.* [34], came from a market in Tororo, Uganda, and had been identified to be commonly eaten by pregnant participants in an ongoing clinical study (NCT00993031) [39]. Participants reported consuming approximately 70 g per day, slightly higher than the 30 g/day modal amount reported elsewhere in the literature [40]. The pure clay mineral smectite (STx-1b*, Clay Minerals Society*) was selected because it is one of the clay minerals that are most commonly found in high proportion in geophagic earths [40], such that its impact on Fe absorption would be highly relevant. The Fe content of the geophagic earth and smectite, as determined by inductively coupled plasma atomic emission spectroscopy (ICP-AES), are 17,475 ppm and 4795 ppm, respectively [34].

The quantity of clay minerals and geophagic earth administered to the chickens was determined based on quantities typically consumed by humans, reported previously [9]. The mean amount of this particular type of geophagic earth consumed daily (~70 g) was converted to a ratio of earth to average body weight of a typical human (~70 kg, or 1:1000). This ratio was then applied to the estimated mean body weight of the chicks to calculate earth/clay doses (Table 2). The earth/clay dose was increased weekly based on the estimated mean body weight of the chicks. To account for the varying levels of clay minerals found in geophagic earth [9,15,26] three treatment groups were designed using the smectite. The amount of smectite gavaged daily was 20%, 35% and 50% of the amount of geophagic earth gavaged (group 5) in groups 2, 3 and 4, respectively.

### 2.2. Study Design and Assessments

At week 4, after hatching, chicks were randomized into five treatment groups (on the basis of body weight, gender and blood Hb concentration) to ensure equal allocation between treatment groups (*n* = 12). Treatment groups were gavaged with varying doses of geophagic earth and smectite clay mineral samples daily for four weeks (Table 2). Gavaging was performed on all birds each morning before they were exposed to food. The earth and clay samples were suspended in water, and oil was applied to the pipettors before gavaging. Treatment group 1 was treated as a control and was gavaged daily with water. At the end of four weeks, birds were euthanized using carbon dioxide. Sections of the duodenum and liver were removed and immediately frozen in liquid nitrogen then stored in a −80 °C freezer until analysis.

Body weight and Hb were measured weekly. Ten birds per treatment group were randomly sampled each week for Hb analysis. Blood samples were collected from the wing vein (~100 µL) using micro-hematocrit heparinized capillary tubes (Fisher, Pittsburgh, PA, USA). Blood Hb concentrations were determined using the cyanmethemoglobin method (H7506-STD, Pointe Scientific Inc., Canton, MI, USA).

#### 2.2.1. Isolation of Total RNA

RNA extraction was performed from 30 mg of the distal duodenal tissue on six randomly selected birds per treatment group using a Qiagen RNeasy Mini Kit (Qiangen Inc., Valencia, CA, USA), according to the manufacturer’s protocol and as previously described [38].

#### 2.2.2. DMT-1, DcytB and Ferroportin Gene Expression Analysis

As previously described [37,38,41], PCR was carried out with primers chosen from the fragment of the chicken (*Gallus gallus*) duodenal DMT1 gene (GeneBank datatbase; GI 206597489), DcytB gene (GI 20380692) and Ferroportin gene (GI 61098365). Ribosomal 18S was used to normalize the results, with primers from the *Gallus gallus* 18S ribosomal RNA (GI 7262899).

All PCR products were separated by electrophoresis on 2% agarose gel, stained with ethidium bromide, and quantified using the Quantity-One 1-D analysis program (Bio-Rad, Hercules, CA, USA).

#### 2.2.3. Liver Ferritin and Fe

Four randomly selected liver samples per treatment group were treated as described elsewhere [37,42]. Briefly, the frozen tissue samples were thawed on ice for approximately 30 min. One gram of sample was diluted into 1 mL of 50 mM Hepes buffer, pH 7.4, and homogenized on ice at 5000 g for 2 min. One mL of each homogenate was subjected to heat treatment for 10 min at 75 °C to aid isolation of ferritin since other proteins are not stable at that temperature [42,43]. After heat treatment, the samples were immediately placed on ice for 30 min. Thereafter, samples were centrifuged at 13,000 g for 30 min at 4 °C until a clear supernatant was obtained and the pellet containing most of the insoluble denaturated proteins was discarded. All tests were conducted in duplicate for each animal.

#### 2.2.4. Electrophoresis and Staining Gels

Native polyacrylamide gel electrophoresis was conducted using a 6% separating gel and a 5% stacking gel. Samples were run at a constant voltage of 100 V. After electrophoresis, the gels were treated with either of the two stains as described earlier [44]: Coomasie blue G-250 stain, specific for proteins, or potassium ferricyanide (K_3_Fe(CN)_6_) stain, specific for Fe. The corresponding band found in the protein and Fe stained gel was considered to be ferritin.

The gels were scanned with Bio-Rad densitometer. Measurements of the bands were conducted using the Quantity-One 1-D analysis program (Bio-Rad, Hercules, CA, USA). The local background was subtracted from each sample. Horse spleen ferritin (Sigma Aldrich Co., St. Louis, MO, USA) was used as a standard for calibrating ferritin protein and Fe concentrations of the samples [37,38,42,43].

### 2.3. Statistical Analysis

One-way ANOVA tests were used to compare mean body weight, feed intake, Fe intake and Fe status indicators between or among treatment groups using JMP^®^ Pro 10.0.0 (SAS Institute Inc., Cary, NC, USA). *Post-hoc* Tukey’s honestly significantly different (HSD) tests were performed to make pairwise comparisons when ANOVA tests indicated significance. Statistical significance was defined as *p* < 0.05.

## 3. Results

### 3.1. Growth Rates, Feed Intakes, Fe Intakes and Hemoglobin (Hb)

There were no significant differences in body weight or weekly cumulative feed intake by treatment group at any time (Table 3). Weekly cumulative Fe intake was significantly higher among birds gavaged with geophagic earth (Group 5) than all other treatment groups at every week (*p* ≤ 0.0001, Table 3). Despite a two- to three-fold higher weekly Fe intake in Group 5, Hb concentrations were not significantly different among any of the groups at any time point (Table 3).

### 3.2. Liver Ferritin and Fe

**Figure 1:**
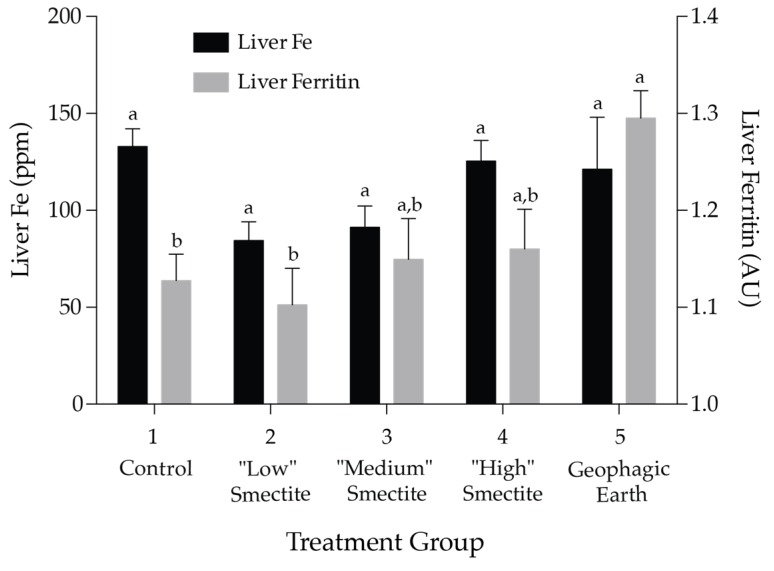
Liver ferritin and liver Fe at day 28. Ferritin levels are means (*n* = 4, ± SEM) and are expressed relative to a standard of horse spleen ferritin as arbitrary units (AU). Liver Fe levels are means (*n* = 10 ± SEM). Means without a common letter are significantly different at *p* < 0.05 (Tukey’s honest significant difference (HSD)).

Mean liver ferritin levels (expressed as relative to a standard of horse spleen ferritin as arbitrary units (AU)) differed significantly as a function of the treatment group, *F* (4, 15) = 4.37, *p* = 0.015 (Figure 1). The geophagic group (Treatment group 5) had significantly higher mean liver ferritin (*M* = 1.295 AU) than treatment groups 1 (*M* = 1.128 AU) and 2 (*M* = 1.103 AU). No other comparisons were significant. Mean liver Fe concentration (ppm) did not differ significantly by treatment group.

### 3.3. Gene Expression of Fe Transporters DMT-1, Ferroportin and DcytB in the Duodenum

**Figure 2:**
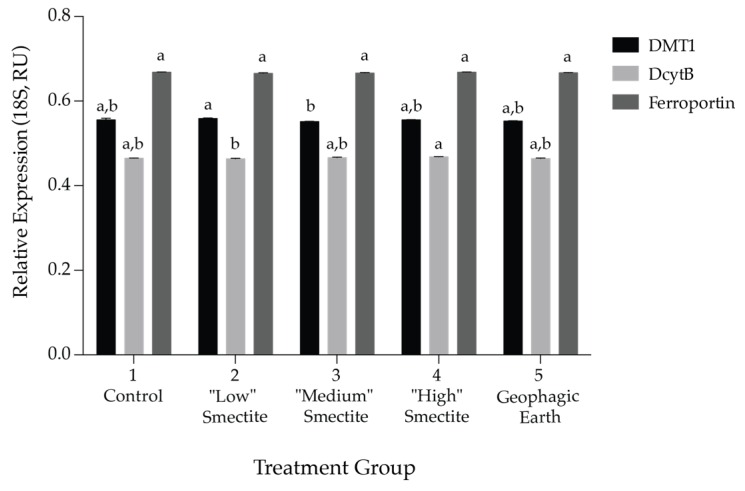
Chicken duodenal mRNA expression of divalent metal transporter 1 (DMT1), duodenal cytochrome B (DcytB), and ferroportin. Expression is shown relative to expression of 18S rRNA in arbitrary units (AU). Values are means ± SD, *n* = 5. Means without a common letter are significantly different at *p* < 0.05 (Tukey’s HSD).

Gene expression analysis, with results reported relative to 18S rRNA, indicated no significant differences between groups in expression of duodenal ferroportin. Relative expression of both DMT1 and DcytB differed significantly as a function of the treatment group (Figure 2, *F* (4, 20) = 2.99, *p* = 0.0435 and *F* (4, 20) = 3.89, *p* = 0.017, respectively). Treatment group 2 (*M* = 0.559 AU) had significantly higher relative DMT1 expression than treatment group 3 (*M* = 0.551 AU) and significantly lower relative DcytB expression (*M* = 0.464 AU) than treatment group 4 (*M* = 0.468 AU). Overall, treatment did not lead to biologically relevant alterations in transcript levels of duodenal transporters, suggesting that these treatments did not drastically alter the birds’ iron status.

## 4. Discussion

Our first objective was to identify and apply an *in vivo* methodology for studying the relationship between geophagy and Fe status. *Gallus gallus* has several advantages over previous *in vitro* models. These include a closer approximation of the digestive process; the presence of mucin and a regular interaction with the food matrix are particular advantages. It also is a fast-growing animal that is sensitive to dietary deficiencies of mineral elements such as Fe. As such, it holds potential as a relevant model and as a source of tissues for *in vitro* Fe bioavailability studies, *in vivo* feeding trials, or both. In addition, chickens are cheaper and easier to maintain than many other species. Moreover, the overall size of the adult broiler allows for repeated blood sampling at volumes suitable for measurement of mineral elements*.* The chicken model also offers advantages over previous *in vivo* work (done in humans) including the possibility of a more rigorous evaluation of Fe status and permitting greater control over geophagic earth dosing via oral gavage of the sample of interest.

Our second objective was to determine the effect of geophagic materials on Fe status, both in terms of their ability to provide bioavailable Fe and their ability to inhibit Fe absorption from other sources. In terms of providing bioavailable Fe, although treatment group 5 received significantly higher amounts of elemental Fe than other groups, no significant difference was observed between Hb, liver Fe concentration or expression of genes involved in Fe absorption and transportation. However, treatment group 5 did have significantly higher liver ferritin levels as compared to the control, indicating increased liver Fe stores in group 5; hence, some Fe in the geophagic earth was bioavailable. Differences in duodenal DMT1 and DcytB between treatment groups were minor, suggesting that all birds were Fe deficient (as expected due to marginal levels of Fe in the experimental diet) and therefore there was a gene expression upregulation to compensate for the lack of Fe in their diets. It also suggests that the treatment did not affect the tissue functionality (*i.e.*, the gene expression) by increasing the dietary Fe bioavailability or increasing Fe content in the lumen (from the soil). These findings are consistent with *in vitro* experiments with smectite (STx-1b*, Clay Minerals Society*) and Ugandan geophagic earth using the Caco-2 model [34], which also found that when combined with a food source of Fe (cooked white beans), these samples do not provide significantly increased amounts of bioavailable Fe.

In terms of geophagic substances’ ability to inhibit Fe absorption, we found no significant negative effects of geophagic earth/clay mineral consumption on Fe status. This indicates that these samples, when dosed at this level, do not have the ability to adsorb dietary Fe in amounts substantial enough to cause a reduction in Fe absorption. This is in striking contrast to previous *in vitro* and *in vivo* studies. Of the three *in vitro* studies to date that have investigated the ability of geophagic substances to bind to bioavailable Fe [20,33,34], most but not all of the samples were shown to reduce bioavailable or bioaccessible Fe. Of the five *in vivo* studies in humans [19,21,22,35,36], four concluded that the consumption of earth prior to the consumption of Fe had the ability to reduce the absorption of Fe. 

Furthermore, our expectation of reduced Fe bioavailability was based on smectite’s well-established adsorptive properties. Smectite clays have been found to bind well to a number of substances (e.g., herbicides [45], tannic acid [46], T-2 toxin [47] and aflatoxins [48]), an adsorptive capacity often attributed to its high cation exchange capacity (CEC). Because of its high CEC and adsorptive capacity, it has been suggested as integral to the ability of geophagic earths to bind Fe [20]. Oral diosmectite has also been found to interact with mucus molecules to strengthen the mucosal barrier in the gastrointestinal tract [47,49] which could potentiate the negative effect of smectite on dietary Fe absorption.

Overall, the consumption of the clay mineral smectite and geophagic earth at concentrations in this study resulted in minimal impact on Fe status. We hypothesize that the most likely explanation for the absence of observed effect on Fe status is inadequate dosing of both smectite and geophagic earth. Our calculations for clay and earth dosing were based on a ratio of geophagic earth to body weight in humans. However, in order to investigate the interaction between the geophagic earth and the Fe in the food, it might have been more appropriate to calculate dosing based on the ratio of daily food consumption to daily geophagic earth. This ratio would result in much larger doses of geophagic earth and clay minerals treatments (~1–7 g/day). Alternatively, it may have been more appropriate to calculate dosages based on the relative length of alimentary canal (human: ~5.5–7 m; chicken: ~2 m) in order to accurately capture the effect of the interaction of geophagic soil or mineral clay with the intestinal brush border. This logic would also result in a much larger dose of geophagic earth and clay mineral (~5–25 g/day).

This study has several additional limitations. The human diet is much more diverse than that fed to these animals, and typically includes both heme and non-heme Fe, such that interactions between geophagic earth and dietary Fe may be more complex that what is modeled with a simple corn-based diet. Further, chickens received earth or clay minerals only once per day and after a night of fasting, but humans have reported consuming earth sometimes multiple times a day and both before and after meals. The resources and expertise needed for animal experimentation must be weighed carefully when considering *in vitro* *vs. in vivo* approaches.

## 5. Conclusions

In summary, *Gallus gallus* represents a significant advance over previous methods for studying the effects of geophagy on Fe status. This versatile model allows not only the investigation of the elemental and bioavailable Fe content but also permits the determination of any inhibitory effects on dietary Fe absorption in a relatively simple and cost-effective way. As such, the use of this model with geophagic and other pica substances will likely lead to important clarifications about the health consequences of geophagy, a nutritional enigma of real public health concern.

## Figures and Tables

**Table 1:** Composition of Fe-inadequate experimental diet.

Ingredient	(g/kg Diet)
Ground yellow corn	750
Dry skim milk	100
DL-Methionine	2.5
Vegetable oil	30
Corn starch	46.5
Choline chloride	0.75
Vitamin premix ^a^	10
Mineral premix ^a^	60
Ferric citrate ^b^	0.25
Total	1000

^a^ Vitamin-mineral premix provided per kg of diet: retinyl palmitate, 1208 µg; ergocalicferol, 5.5 µg; DL-α-tocopheryl acetate, 10.72 mg; menadione, 0.5 mg; D-biotin, 0.05 mg; choline chloride, 0.5 g; folic acid, 0.3 mg; niacin, 15 mg; Ca-D pantothenate, 10 mg; riboflavin, 3.5 mg; thiamin, 1 mg; pyridoxine, 1.5 mg; cyanocobalamin, 17.5 µg; CuSO_4_*5H_2_O, 6 mg; C_2_H_8_N_2_*2HI, 0.14 mg; MnO, 4 mg; Na_2_SeO_3_, 0.3 mg; and ZnO, 100 mg; ^b^ Ferric citrate was marginally lower than NRC recommendations so that Fe status of birds would be more sensitive to different Fe intakes.

**Table 2:** Dosage of clay minerals and geophagic earth, by treatment group and week of experiment.

Treatment Group		Dosages g of Clay Minerals or Geophagic Earth Gavaged/Day (mg Fe Content)				
		1 (*n* = 12)	2 (*n* = 12)	3 (*n* = 12)	4 (*n* = 12)	5 (*n* = 12)
Week	Predicted Mean Chicken Mass (kg)	Control ^a^	“Low” Smectite	“Medium” Smectite	“High” Smectite	Geophagic Earth
1	0.20	0	0.04 (19)	0.07 (34)	0.10 (48)	0.20 (3.50)
2	0.30	0	0.06 (29)	0.10 (48)	0.15 (72)	0.30 (5.24)
3	0.30	0	0.06 (29)	0.10 (48)	0.15 (72)	0.30 (5.24)
4	0.35	0	0.07 (34)	0.12 (58)	0.17 (82)	0.35 (6.12)

^a^ Control was gavaged with water.

**Table 3:** Body weights, cumulative weekly feed intake, Fe intake, and Hb from day 0 to day 28 of experiment, by treatment group.

Treatment		Day 0	Day 7	Day 14	Day 21	Day 28
Body weight (g) ^1^						
1	Control	210 ^a^	243.1 ^a^	354.0 ^a^	453.4 ^a^	520.4 ^a^
2	Low Smectite	209 ^a^	241.6 ^a^	361.1 ^a^	480.0 ^a^	534.2 ^a^
3	Medium Smectite	210 ^a^	233.6 ^a^	344.6 ^a^	443.0 ^a^	494.7 ^a^
4	High Smectite	210 ^a^	232.3 ^a^	342.2 ^a^	419.8 ^a^	458.8 ^a^
5	Geophagic Earth	211 ^a^	250.8 ^a^	343.4 ^a^	441.2 ^a^	503.5 ^a^
Feed intake (g/week/bird) ^1,2^						
1	Control	-	203.4 ^a^	293.9 ^a^	346.3 ^a^	438.8 ^a^
2	Low Smectite	-	189.3 ^a^	276.6 ^a^	362.9 ^a^	382.3 ^a^
3	Medium Smectite	-	183.7 ^a^	261.1 ^a^	308.9 ^a^	392.3 ^a^
4	High Smectite	-	183.2 ^a^	268.7 ^a^	324.9 ^a^	361.8 ^a^
5	Geophagic Earth	-	181.9 ^a^	279.9 ^a^	307.4 ^a^	380.2 ^a^
Fe intake (mg/week/bird) ^1,2,3^						
1	Control	-	10.2 ^a^	14.7 ^a^	17.3 ^a^	21.9 ^a^
2	Low Smectite	-	10.8 ^a^	15.8 ^a^	20.1 ^a^	21.4 ^a^
3	Medium Smectite	-	11.5 ^a^	16.5 ^a^	19.5 ^a^	23.7 ^a^
4	High Smectite	-	12.4 ^a^	18.1 ^a^	20.9 ^a^	23.9 ^a^
5	Geophagic Earth	-	33.6 ^b^	50.7 ^b^	52.1 ^b^	61.8 ^b^
Haemoglobin (g/dL) ^4^						
1	Control	9.02 ^a^	8.48 ^a^	8.37 ^a^	8.30 ^a^	8.28 ^a^
2	Low Smectite	9.02 ^a^	8.55 ^a^	8.40 ^a^	8.21 ^a^	8.16 ^a^
3	Medium Smectite	9.02 ^a^	8.43 ^a^	8.28 ^a^	8.25 ^a^	8.18 ^a^
4	High Smectite	9.02 ^a^	8.42 ^a^	8.24 ^a^	8.20 ^a^	8.18 ^a^
5	Geophagic Earth	9.02 ^a^	8.44 ^a^	8.37 ^a^	8.30 ^a^	8.19 ^a^

^a,b^ within a column, means without a common letter are significantly difference, *p* < 0.05 (Tukey’s honest significant difference (HSD)); ^1^ Values are means, *n* = 12, except where noted; ^2^ Values are weekly feed/Fe intakes for the seven days preceding the day designated in the column heading; ^3^ Fe intakes were calculated by summing Fe derived from feed intake data (50 ppm) with Fe derived from dosed geophagic earth (17,475 ppm) or mineral clay (4795 ppm). Fe concentration of the geophagic earth and smectite were determined using inductively-coupled plasma atomic emission spectroscopy (ICP-AES); ^4^ Values are means, *n* = 10.

## Abbreviations

The following abbreviations are used in this manuscript:

Fe	iron
Hb	hemoglobin
TEC	total elemental composition
DMT1	divalent metal transporter 1
DcytB	duodenal cytochrome B
CEC	cation exchange capacity
GI	gastrointestinal
ICP-AES	inductively-coupled plasma atomic emission spectroscopy
